# Homozygous nonsense mutation of *WNT10B* gene in a Moroccan family with split-hand foot malformation identified by exome sequencing: a case report

**DOI:** 10.11604/pamj.2021.39.21.26176

**Published:** 2021-05-07

**Authors:** Siham Chafai Elalaoui, Nawfal Fejjal, Yun Li, Holger Thiele, Janine Altmüller, Soukaina Guaoua, Peter Nürnberg, Bernd Wollnik, Abdelaziz Sefiani, Ilham Ratbi

**Affiliations:** 1 Génomique et Epidémiologie Moléculaire des Maladies Génétiques (G2MG), Centre GENOPATH, Faculté de Médecine et de Pharmacie, Mohammed V University in Rabat, Rabat, Maroc,; 2 Département de Génétique Médicale, Institut National d’Hygiène, Rabat, Maroc,; 3 Service de Chirurgie Plastique Pédiatrique, Hôpital des Enfants, Centre Hospitalier Universitaire Ibn Sina, Faculté de Médecine et de Pharmacie, Université Mohammed V, Rabat, Maroc,; 4 Center for Molecular Medicine Cologne (CMMC), University of Cologne, Cologne, Germany,; 5 Institute of Human Genetics, University Hospital Cologne, University of Cologne, Cologne, Germany,; 6 Cologne Excellence Cluster on Cellular Stress Responses in Aging-Associated Diseases (CECAD), University of Cologne, Cologne, Germany,; 7 Cologne Center for Genomics, University of Cologne, Cologne, Germany

**Keywords:** Homozygous mutation, WTN10B gene, split-hand foot malformation, case report

## Abstract

Split-hand foot malformation (SHFM) is a clinically heterogeneous congenital limb defect affecting predominantly the central rays of hands and/or feet. The clinical expression varies in severity between patients as well between the limbs in the same individual. SHFM might be non-syndromic with limb-confined manifestations or syndromic with extra-limb manifestations. Isolated SHFM is a rare condition with an incidence of about 1 per 18,000 live born infants and accounts for 8-17 % of all limb malformations. To date, many chromosomal loci and genes have been described as associated with isolated SHFM, i.e., SHFM1 to 6. SHFM6 is one of the rarest forms of SHFM, and is caused by mutations in WNT10B gene. Less than ten pathogenic variants have been described. We have investigated a large consanguineous Moroccan family with three affected members showing feet malformations with or without split hand malformation phenotypes. Using an exome sequencing approach, we identified a homozygous nonsense variant p.Arg115* of WNT10B gene retaining thereby the diagnosis of SHFM6. This homozygous nonsense mutation identified by exome sequencing in a large family of split hand foot malformation highlights the importance of exome sequencing in genetically heterogeneous entities.

## Introduction

Split-hand foot malformation (SHFM) is a clinically and genetically heterogeneous condition characterized by congenital limb defect affecting predominantly the central rays of hands and/or feet [1]. The clinical expression varies in severity between patients as well between the limbs in the same individual [2,3]. SHFM might be non-syndromic with limb-confined manifestations or syndromic with extra-limb manifestations [4]. Isolated SHFM is a rare condition with an incidence of about 1 per 18,000 live born infants and accounts for 8-17% of all limb malformations [5]. To date, seven chromosomal loci associated with isolated SHFM have been described, i.e., SHFM1 to 6. Most of the known SHFM loci are associated with chromosomal rearrangements that involve small deletions or duplications of the human genome. In addition, five genes, i.e., TP63 (tumor protein p63, MIM 603273), *WNT10B* (awingless-type MMTV integration site family, member 10B, MIM 601906), *DLX5 (distal-less homeobox*5, MIM 600028), ZAK (*leucine zipper- and sterile alpha motif-containing kinase*, MIM 609479), and CDH3 (*cadherin*3, MIM 114021), are known to carry point mutations in patients affected by SHFM [1]. The autosomal dominant mode of inheritance is typical for SHFM1 (DLX5 gene on 7q21), SHFM3 on 10q24, SHFM4 (*TP63* gene on 3q28) and SHFM5 on 2q31 [6-12]. Two autosomal recessive forms have been reported, i.e., SHFM1D associated with deafness, due to DLX5 mutations and SHFM6 due to *WNT10B* mutations [9,12,13]. In addition, SHFM2 has been assigned to Xq26 by linkage analyses in a large Pakistani kindred [4]. In this study, using an exome sequencing approach, we identified a homozygous nonsense *WNT10B* mutation, in a large consanguineous Moroccan family with three affected members showing feet and split hand malformations malformation phenotypes, confirming the diagnosis of SHFM6.

## Patient and observation

**Figure 1:**
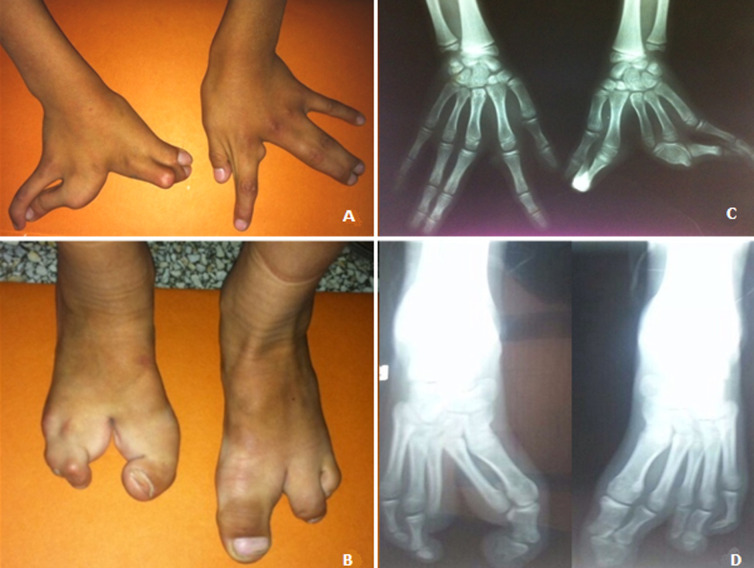
clinical (A, B) and radiological (C, D) features of SHFM phenotypes observed in patient II: 6

**Clinical presentation**: the propositus II: 6, a twelve years-old female, was referred to our institute for a medical genetic consultation with a chief complaint of malformations. She was the sixth liveborn child of a healthy consanguineous couple from an isolated village of the South of Morocco. Her parents reported normal psychomotor development. On clinical examination, she had very mild facial dysmorphism including flat nasal bridge, down slanting palpebral fissures, short proeminent philtrum, high arched palate and crowded teeth. She showed syndactylies and clefts on both hands and feet. On the right hand, she had cleft hand deformity, syndactyly of the thumb and second finger, absence of the third finger, and a deformity of the proximal phalanx of the 4^th^ finger. On the left hand, she had a radial deviation of the thumb and second finger, partial cutaneous syndactyly of the thumb and second finger, cutaneous syndactyly of the third and fourth fingers, and an ulnar deviation of the third, fourth and fifth fingers (Figure 1A). On the right foot, she had cleft foot, with a broad hallux and apparently absence of the second toe, cutaneous syndactyly of the fourth and fifth toes, and absence of the third toe. There was also a cutaneous syndactyly of the first and second metatarses, on one part and on the other part, cutaneous syndactyly of the third, fourth and fifth metatarses. On the left foot, she had a cleft foot, with a very broad hallux, concording with a syndactyly of the hallux and the second toe, and cutaneous syndactyly of the third and fourth toes (Figure 1B). She did not have other somatic abnormalities.

Radiography showed on right hand, a cleft hand deformity, with deformity of the second and third phalanges of the second finger, absence of the third finger, with ulnar deviation of the fourth and fifth fingers. On left hand, radiography showed radial deviation of the thumb and the second finger (Figure 1C). Radiography of the right foot showed osseous syndactyly of the hallux and the second toe, absence of the third toe. Radiography of the left foot showed fusion of the second phalanges of the hallux and second toe, absence of the second phalange of the third toe (Figure 1D). Radiography of upper and lower limbs was normal. Cardiac, abdominal and pelvic ultrasonographies were normal. Two other older affected siblings, a female of 25 and a male of 18 had isolated cutaneous syndactyly of toes without involvement of hands or other malformations. Parents did not show any hand or foot malformation, and their teeth examinations were normal.

**Figure 2:**
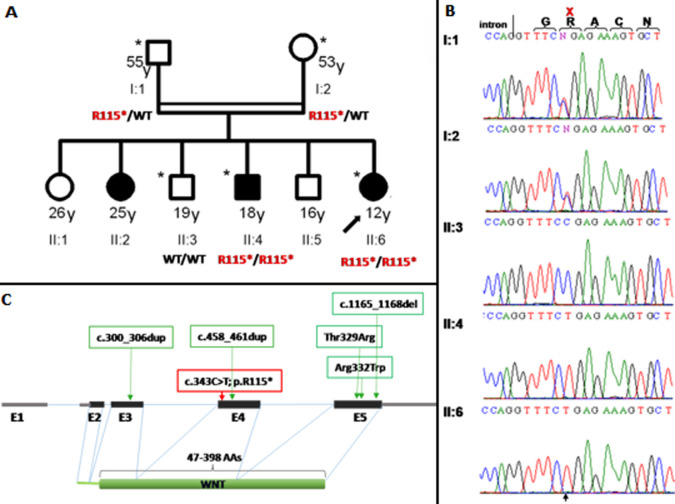
A) pedigrees of the studied family, affected individuals are shaded, the proband is indicated by an arrow; investigated members by WES and Sanger sequencing are with an asterisk; B) electropherograms of identified homozygous *WNT10B* mutation; two patients (II: 4 and II:6) present homozygous R115 and both parents are heterozygotes (I: 1 and I: 2); one health brother (II: 3) shows homozygous wild-type; C) schematic view of the *WNT10B* gene structure, protein domains, and localization of identified *WNT10B* mutations; the mutations reported were indicated with green frame and the mutation identified in this study was indicated with red frame

**Genetic analysis**: all patients or their legal representatives gave written informed consent to the study, which was performed in accordance with the Declaration of Helsinki protocols and approved by the local institutional review boards. Deoxyribonucleic acid (DNA) was isolated from peripheral blood for all patients using standard techniques [14]. Complete DNA Sanger sequence analysis of the entire coding region and flanking introns of the LRP4 gene was performed in the proband DNA sample on an ABI3730 DNA sequence (Applied Biosystems, Foster City, CA, USA) as reported previously [15]. Whole Exome Sequencing (WES) was undertaken for individuals I: 1, I: 2, II: 6 (Figure 2A). DNA enrichment for WES was achieved using Nimble GenSeqCap EZ Human Exome Library v2.0. Paired-end sequencing (100bp) was run on an IlluminaHiSEQ2000. Sanger sequencing of all 38 exons of LRP4 gene did not identify any putative mutation. In data analysis of WES, when we selected biallelic variants or previously reported pathogenic non-synonymous, frameshift, nonsense or splice site variants consistent with recessive inheritance that co-segregated with the disease phenotype, we identified putative causing variant in *WNT10B* (MIM 601906; NM_003394.3). It was a homozygous nonsense variant R115* in exon 4. The resultant transcript was assumed to lead to the expression of a truncated protein. It was confirmed by Sanger sequencing, predicted to be damaging and segregated with the disease phenotype, such that all affected individuals were homozygous, whereas unaffected parents were heterozygous and unaffected sibling II : 3 was homozygous for the wild type (Figure 2A, Figure 2B).

## Discussion

Split-hand/split-foot malformation (SHFM), representing variable degree of median clefts of hands and feet, is a genetically heterogeneous group of limb malformations with seven loci mapped on different human chromosomes. However, only 3 genes (*TP63, WNT10B, DLX5*) for the seven loci have been identified [1]. Phenotypic variability has been reported not only among families but also among affected members within the same family. In few cases, even within the same individual one extremity was found more affected than the other [1]. SHFM6 type 6 is inherited as an autosomal recessive manner. This entity is due to mutations in *WNT10B* gene [13]. Twelve affected members from a large Turkish family exhibiting autosomal recessive inheritance for SHFM were firstly clinically described [16]. All patients had central feet reductions with or without hand involvement, except one individual who had just unilateral hand syndactyly with no foot involvement. Six years later, this family was enrolled in a homozygosity mapping study. It leads to the identification of a novel SHFM locus at 12q13.11-q13 and by subsequent candidate gene approach a homozygous missense *WNT10B* mutation (p.Arg332Trp) [13].

This homozygous mutation was interestingly also found in one unaffected individual from the same family, whereas the patient with atypical SHFM was not carried. To explain the observed phenotypes in this family, authors assumpt that maybe a *WNT10B* mutation is necessary, but not sufficient to produce SHFM. The second reported case was a sporadic Swiss patient with a homozygous4-bp duplication resulting in a premature termination codonin *WNT10B* gene. Nine heterozygous relatives showed no sign of SHFM [17]. This case supported that sporadic SHFM is not always a dominant trait and that testing for mutations in *WNT10B* should be also considered for these patients. In 2012, a third novel homozygous c.986C>G, p.Thr329Arg mutation in *WNT10B* gene, was found in a large consanguineous Pakistani family with autosomal recessive SHFM phenotype involving hands and feet, and appearing in two generations [18]. Recently, three other consanguineous Pakistani families segregating autosomal recessive SHFM, with phenotypic variability grading from mild preaxial to post-axial involvement as simple syndactyly to severe central clefting of the autopods were reported. Genotyping microsatellite markers and mutation screening of *WNT10B* by Sanger DNA sequencing identified a homozygous 4-bp deletion (c.1165_1168delAAGT) in one family and a homozygous 7-bp duplication (c.300_306dupAGGGCGG) in the two others [19].

To the best of our knowledge, the Moroccan family reported here is therefore the seventh case in the literature with SHFM6 (Figure 2C). We were firstly clinically oriented to a Cenani-Lens syndrome because of the slight facial dysmorphism of the proband and the autosomal recessive segregation associated to complex syndactyly of hands and feet, even if she presented no malformations of the forearm bones. The other affected siblings did exhibit neither relevant dysmorphy nor anomalies of the forearm bones. Sanger sequencing of *LRP4* gene did not identify any variant. Lastly, exome sequencing was performed because of the higher clinical and genetic heterogeneity of this type of limb disorders and leads to define the genetic basis in this family. The identified homozygous WTN10B mutation p. R115* was previously reported in heterozygous state in a patient with Split hand foot [20].

## Conclusion

In conclusion, we report on the first clinical and molecular description of a Moroccan family with autosomal recessive SHFM. This diagnosis allowed us to provide an appropriate course of management to patients, and offer genetic counseling to the family.
